# Pollinator scarcity drives the shift to delayed selfing in Himalayan mayapple *Podophyllum hexandrum* (Berberidaceae)

**DOI:** 10.1093/aobpla/plt037

**Published:** 2013-08-26

**Authors:** Ying-Ze Xiong, Qiang Fang, Shuang-Quan Huang

**Affiliations:** 1 State Key Laboratory of Hybrid Rice, College of Life Sciences, Wuhan University, Wuhan 430072, China; 2 College of Agriculture, Henan University of Science and Technology, Luoyang, Henan 471003, China; 3 College of Life Sciences, Central China Normal University, Wuhan 430079, China

**Keywords:** Autogamy, Baker's law, petal movement, *Podophyllum hexandrum*, *Podophyllum peltatum*, pollinator limitation, self-compatible, stigmatic pollen load.

## Abstract

Recent molecular phylogenetics have indicated that American mayapple (mainly self-incompatible, SI) and Himalayan mayapple, which was considered to be self-compatible (SC), are sister species with disjunct distribution between eastern Asia and eastern North America. We test a hypothesis that the persistence of this early spring flowering herb in the Himalayan region is attributable to the transition from SI to SC, the capacity for selfing in an unpredictable pollination environment. Pollinator observations were conducted in an alpine meadow with hundreds of Himalayan mayapple (*Podophyllum hexandrum* Royle) individuals over 2 years. To examine autogamy, seed set under different pollination treatments was compared. To clarify whether automatic self-pollination is achieved by movement of the pistil as a previous study suggested, we measured incline angles of the pistil and observed flower movement during anthesis using video. Floral visitors to the nectarless flowers were very rare, but solitary bees and honeybees could be potential pollinators. Seed set of bagged flowers was not significantly different from that of open-pollinated, self- or cross-pollinated flowers. However, removal of petals or stamens lowered seed yield. The angles of inclination of pistils did not change during the process of pollination. Automatic self-pollination was facilitated by petals closing and stamens moving simultaneously to contact the stigma. Stigmatic pollen load increased little during the day time, in contrast to a sharp increase when the flowers closed during the night-time. These observations indicated that Himalayan mayapple was SC and delayed self-pollination was facilitated by the movement of petals rather than the pistil. Compared with SI American mayapple, no obvious inbreeding depression in SC Himalayan mayapple may contribute its existence in the uplifting zone. A scarcity of pollinators may have driven the shift to delayed selfing in *P. hexandrum*.

## Abstract (precis)

American mayapple and Himalayan mayapple are a pair of sister species with disjunct distribution between eastern Asia and eastern North America, which the former was considered to be self-incompatible but the later to be self-compatible. We are interested in the diversification of breeding systems in the two species, particularly a usual mode of self pollination in Himalayan mayapple (*Podophyllum hexandrum*) which is achieved by movement of the pistil as a previous study suggested. By contrast, our observations and flower manipulations show that delayed selfing was facilitated by petals closing and stamens moving simultaneously to contact the stigma, and bees were effective pollinators although they were few.

## Introduction

The transition of the breeding system from outcrossing to selfing has been considered to be a widespread evolutionary trend in flowering plants (Stebbins 1957). Baker (1955) noted that, unlike the situation in self-incompatible (SI) species, self-compatibility permits a species to colonize in a new habitat after long-distance dispersal. Darwin (1862) realized that autonomous self-pollination could be an adaptation to reproduction if pollinator service was lost or extremely unpredictable. For example, he illustrated that a nectarless orchid (*Ophrys apifera*) could be favoured by deceit pollination by bees, but was capable of self-fertilization by curving of the caudicles that delivered pollinia directly onto the stigma. Autonomous self-pollination has been classified into three types, termed ‘prior’, ‘competing’ and ‘delayed’, according to the timing of self-pollination relative to cross-pollination (Lloyd and Schoen 1992). Only the last situation, in which self-pollination is delayed until the opportunity for outcrossing has passed, is considered to provide reproductive assurance (Lloyd 1992).

Phylogenetic analysis indicated that the two mayapple species represent an outstanding example of disjunct distribution between eastern Asia and eastern North America (EA-ENA) (Liu *et al.* 2002; Wang *et al.* 2007). Himalayan mayapple, *Podophyllum hexandrum* Royle (Berberidaceae), is an alpine herb occurring in the eastern Himalayas, including southwest China and nearby countries. It was once treated as *Sinopodophyllum hexandrum* (Royle) Ying, a monotypic genus, based on morphological characteristics (e.g. pollen in tetrads) and breeding systems different from American mayapple (*P. peltatum*) (Ying 1979), and it is sometimes regarded as a separate family Podophyllaceae (reviewed in Liu *et al.* 2002). However, molecular phylogeny on Berberidaceae and its relatives showed that Himalayan and American mayapple are sister species and its diversification evolved around 7 (Liu *et al.* 2002) or 6 million years ago (Wang *et al.* 2007). The time of diversification coincides with the rapid uplift of the Himalayas (An *et al.* 2001), suggesting that extant populations of Himalayan mayapple were elevated to the alpine zone, unlike American mayapple. Given that *Dysosma*, a sister to the clade containing American and Himalayan mayapple (Liu *et al.* 2002; Wang *et al.* 2007; Guan *et al.* 2010), is basically SI, it is safe to deduce that the capacity for self-fertilization in Himalayan mayapple evolved recently from SI ancestors. Meanwhile, a shift from SI to self-compatible (SC) was also observed in a few populations of American mayapple where pollinators were extremely rare (Policansk 1983; Whilser and Snow 1992; Crants 2008). This parallel evolutionary transition in the two species of EA-ENA disjunct distribution suggests an ideal group to address the causes of diversification in breeding systems.

In general, the degree of self-pollination within a flower is modified either by spatial or temporal separation of the mature anthers and stigmas or by combinations of these (Moore and Lewis 1965; Karron *et al.* 1997; Kalisz *et al.* 1999). Previous studies on Himalayan mayapple showed that no pollinators visited it, and flowers were capable of automatic self-pollination by inclining the pistil, pushing the stigma into contact with the anthers, after which the pistil returns to its normal upright position at the centre of the flower (Xu *et al.* 1997). It remains unclear in this species whether autogamy is delayed selfing or whether there are any pollinators. The movement of the pistil could involve much higher energetic cost than stamen (or petal) movement, given that stamens are much smaller and more flexible. If this type of self-pollination is really facilitated by the pistil inclining, it suggests that the stigma contacts only one or two anthers in this monogynous flower, leaving other anthers untouched. This mode of pistil movement resulting in selfing is questionable, but is often cited (Ma *et al.* 1997; Shaw 2002; Wang *et al.* 2007; Li *et al.* 2011; Ruan and Teixeira da Silva 2011).

To better understand diversification of the breeding system of sister species with a disjunct distribution between EA-ENA, we conducted a study of the pollination ecology and breeding system of Himalayan mayapple (*P. hexandrum*). We address the following questions: (i) Are there potential pollinators for this nectarless early spring flowering herb in the Himalayan region? (ii) When and how does the autogamy take place? In particular, we try to quantify whether the selfing is caused by the pistil inclining, as previous studies suggested. We examined pollen loads on stigmas at different times of anthesis and movement of flower components, including the petals, stamens and pistil. This investigation permits us to compare breeding systems between the two mayapples and trace diversification of the species under geological disjunction.

## Methods

### The study species

**Figure 1.**
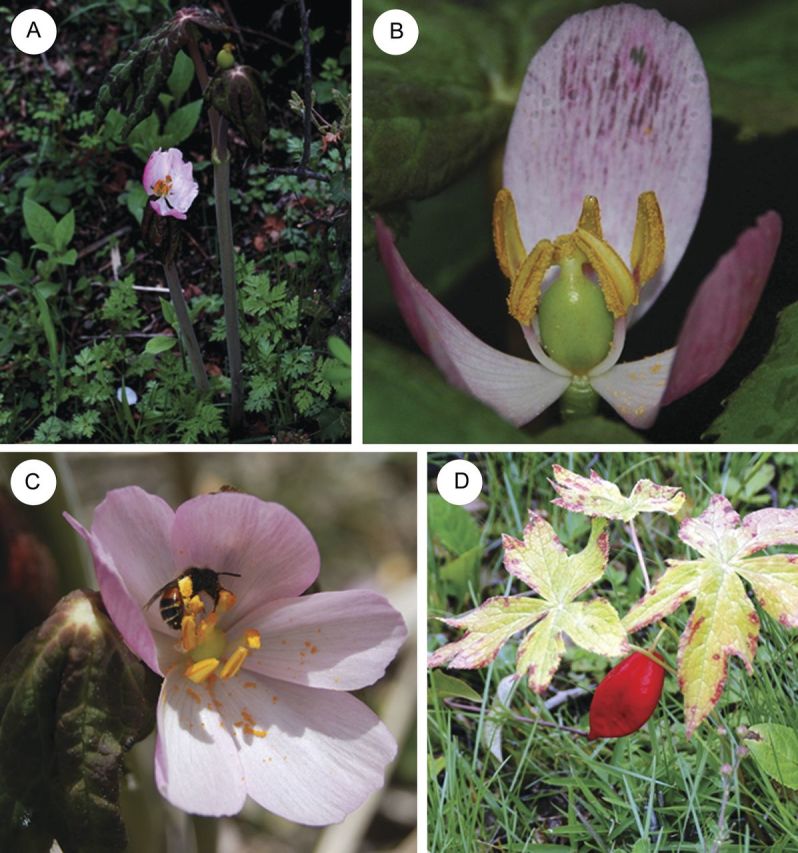
Flowers of Himalayan mayapple (*P. hexandrum*) and pollinators. (A) One plant with two stems each with a terminal flower. (B) Two dehisced anthers touching the stigma in a second-day flower in the late afternoon. (C) A halictid bee collecting pollen*.* (D) A red mature fruit in summer.

*Podophyllum hexandrum* is an alpine herb occurring in the Himalayan region at elevations of 2400–4000 m above sea level (a.s.l.) (Ying *et al.* 2011). It grows in shady and moist plateaus (Fig. 1). Its perennating organ is the underground rhizome from which the aerial part develops during winter. Each stem bears two palmately divided leaves and a single terminal flower bud which flowers in early May; it is one of the early flowering species in the alpine area. The bowl-shaped flower is solitary and has no nectar. Each ﬂower generally has six petals, six stamens and one pistil (ca. 12 mm) with a short style (1–3 mm) and one unilocular ovary with 50–150 ovules (Xu *et al.* 1997; Rajkumar and Ahuja 2010). Stamens dehisce soon after the flower opens and release pollen in tetrads. The flowers last for 4–5 days, flower colour changing from light to dark pink. The fruit matures in August. Seed production depends on pollination and there is no parthenogenesis (Ma *et al.* 1997; Xu *et al.* 1997). Our field work was conducted in a large population with hundreds of *P. hexandrum* individuals around Shangri-La Alpine Botanical Garden (27°54′5″N, 99°38′17″E, 3300–3350 m a.s.l.), Yunnan Province, southwest China. We chose to study the species in that place because the unusual mode of autogamy was observed there (Xu *et al.* 1997).

Flower-visiting insects were observed along transects among four patches, taking 50 min per round. We also observed a fixed sample of about 20 flowers for a period of 30 min at a time. At total of 60 h on 18 sunny days during May 2010 and 2011 were spent watching flower-visiting insects.

### Pollination treatment

To examine the breeding system in *P. hexandrum*, we randomly selected 100 flower buds, labelled them in five groups (20 buds for each group) and bagged each one with a fine nylon mesh net to exclude flower visitors before treatments. The five treatments were as follows: (i) flowers were bagged during anthesis to exclude pollinators, to examine possible automatic self-pollination; (ii) flowers were hand-pollinated with self-pollen to examine self-compatibility; (iii) flowers were hand-pollinated with pollen from other plants at least 5 m away (outcrossing); (iv) stamens were removed from open-pollinated flowers; (v) petals were removed from open-pollinated flowers. These treatments permit us to examine the role of pollinators, and the role of petal closing and stamens in facilitating self-pollination. We compared seed set, which was the ratio of seeds to ovules per flower, among different treatments and from 20 randomly collected open-pollinated individuals (controls) using one-way ANOVA and least significant difference (LSD) post hoc tests.

### Floral movement

#### The opening index of petals and stamens, and movement of the ovary

To quantify whether autogamy in *P. hexandrum* is accomplished through inclining of the pistil, which is then erected again after pollination (Xu *et al.* 1997), we took photographs of 20 flowers from when they had just opened (unpollinated) until the day after they had been pollinated. Using Image-Pro Plus software (Media Cybernetics, Silver Springs, MD, USA), we could obtain the angle of inclination of the pistil before and after pollination. The angle of inclination was measured between the central axis of the ovary and a horizontal line through the base of the ovary. One-way ANOVA was performed to compare the angles per flower pre- and post-pollination. Meanwhile, we used a video focusing on five flowers to record flowering behaviour.

To describe movement of the sexual organs, we introduced an opening index of petals and stamens. We measured the distance between the tips of two opposite petals and stamens, and the diameter of flower buds, using a digital calliper. The distance divided by the diameter was defined as the opening index of petals and stamens. The distances were measured from 10:00 to 16:00 h every half an hour in 15 newly opened flowers and corresponding diameters were measured in the early morning.

### Stigmatic pollen load

To examine when automatic self-pollination occurs during anthesis, pollen grains deposited on stigmas were counted at different flowering stages. We observed that flowers of *P. hexandrum* opened in the morning (around 10:00 h) and closed in late afternoon (after 16:00 h) every day during anthesis. Sixty unopened flowers were divided into four groups, and then we collected the stigmas at four different flowering times, i.e. 17:00 h on the first day, 9:00 h on the second day, 17:00 h on the second day and 9:00 h on the third day. Fifteen stigmas from each collection were fixed in standard FAA solution (formalin : acetic acid : alcohol = 90 : 5 : 5 by volume) in separate microcentrifuge tubes, and pollen grains on each stigma were counted under a microscope. Because stigmatic pollen counts were not normally distributed, they were analysed using non-parametric statistics (Mann–Whitney *U* test) for pairwise comparisons among four times of collection at the 0.05 level of significance. All data analysis was conducted in SPSS, version 17.0. All means are presented with standard errors (±SE).

## Results

### Pollinator observations

Floral visitors to Himalayan mayapple were very rare. Our 60-h observation recorded only a total of 27 visits by solitary bees (*Halictus*, nine visits), honeybees (*Apis cerana*, four visits), sepsid flies (Sepsidae, six visits), soldier flies (Stratiomyidae, four visits) and heteropteran bugs (Coreidae, Lygaeus and Miridae, four visits). Except for *Halictus* and honeybees, flies and other small insects could not serve as pollinators, because no pollen grains were seen on their bodies. In addition, we also noted 19 visits by tiny insects (thrips) foraging pollen, but they did not touch the stigmas. These observations indicated that bees were potential pollinators, but they rarely visited this nectarless herb (Fig. 1).

### Breeding system

**Figure 2.**
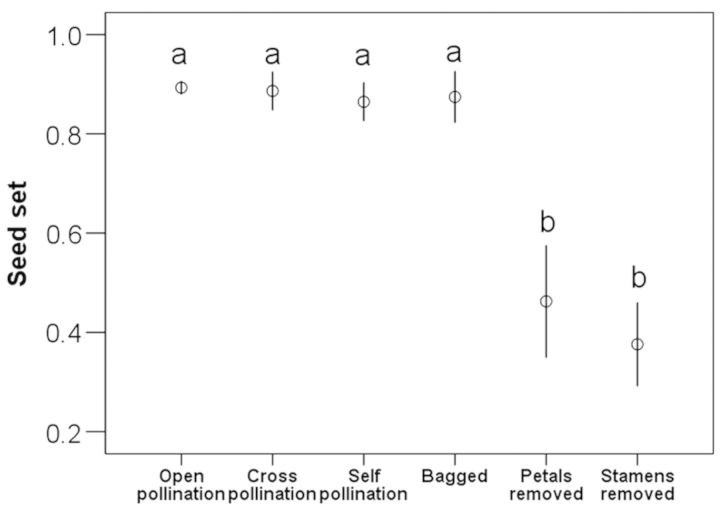
Comparison of seed set per fruit (mean ± SE) under six pollination treatments (open pollination, hand cross-pollination, hand self-pollination, flower bagged, all petals removed and all stamens removed). One-way ANOVA, *F*_5,109_ = 16.549, *P* < 0.001, significant differences between means are indicated by the letters that differ by LSD's multiple comparison test.

Seed sets of bagged flowers (autogamy) and open-pollinated flowers were high and not significantly different from each other or from hand cross- or self-pollination treatments (Fig. 2). These results indicated that Himalayan mayapple was fully SC and capable of automatic self-pollination. Seed sets after the removal of petals and stamens were not significantly different from each other, but they were significantly lower than that of control flowers (*F*_5,109_ = 16.549, *P* < 0.001; Fig. 2). The finding of seed sets, albeit relatively low, in emasculated flowers indicated that these flowers did receive pollen from other flowers, suggesting potential insect pollination. The finding of low seed set from autogamy in petal-removed flowers suggested that the petals facilitated automatic self-pollination in this species.

### Pistil, stamen and petal movement

In contrast to previous observations, we observed that the pistils did not move during anthesis. The angle of inclination of the pistils at flower opening (71.27 ± 7.76°) was not significantly different from that at a late stage of anthesis (71.45 ± 7.32°) (*F*_1,41_ = 0.006, *P* = 0.937). The pistils did not move during flowering, being inclined from the beginning.

**Figure 3.**
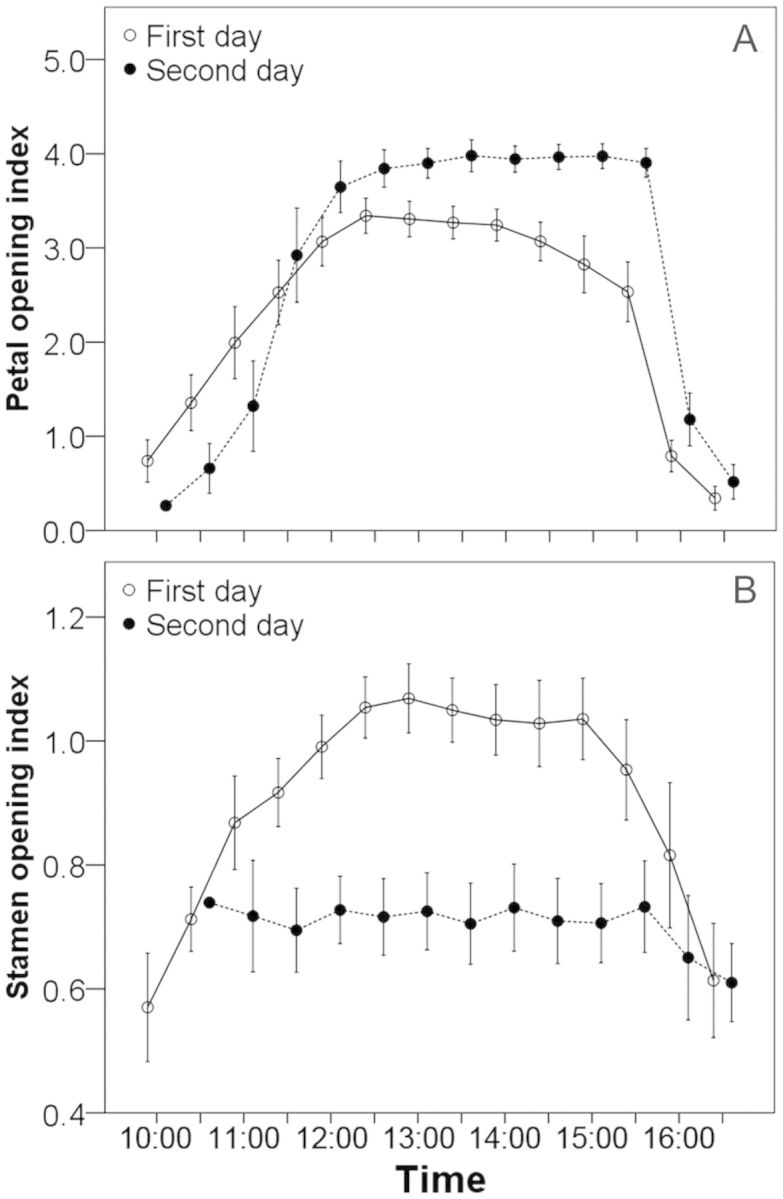
Diagram of the petal opening index (A, mean ± SE) and the stamen opening index (B, mean ± SE) on the first day (open circles) and second day (solid circles) in *P. hexandrum* flowers. The time interval is 30 min.

Petals regularly opened around 10:00 h in the morning and closed after 16:00 h on every fine day during anthesis. When flowers opened, stamens began to extend outwards following the petal movement. When petals began to close, stamens were forced close to the pistil and consequently pollen from dehisced anthers touched the stigma, resulting in self-pollination (Fig. 3). This observation corresponded to the change in the opening index of the petals and stamens, which both began to extend gradually at 10:00 h, reached a peak at 12:00 h and then decreased to nearly zero again at about 16:00 h (Fig. 3). The movement pattern of petals was similar on the first and second days. However, for stamens, the opening index of the second-day flower reached a lower plateau between 11:00 and 15:00 h rather than a peak as in the first-day flower (Fig. 3).

### Stigmatic pollen load

**Figure 4.**
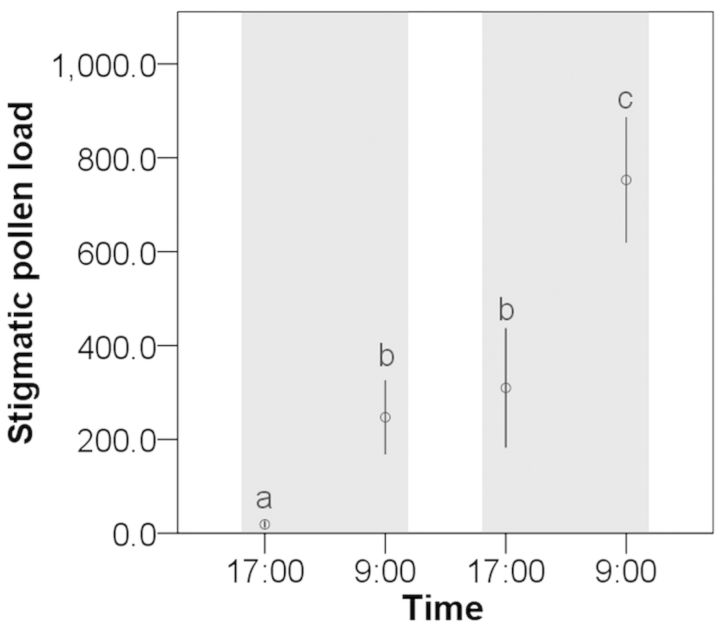
Stigmatic pollen load (mean ± SE) on three consecutive days. Measurement was at 17:00 h on the first day, 9:00 h on the second day, 17:00 h on the second day and 9:00 h on the third day. Significant differences between means are indicated by different letters (Mann–Whitney *U* test). The grey areas mark periods of flower closure.

The pollen grain counts on the stigma increased significantly during two different periods of anthesis. Pollen deposition was low in flowers on the first day of opening (18.9 ± 7.5 grains), but it increased significantly after flowers had experienced one night's closure (247.1 ± 79.1, Mann–Whitney *U* test, *Z* = −3.16, *P* < 0.001). The same situation of a rapid increase in pollen loads occurred during flower closure on the second evening of anthesis (*Z* = −2.96, *P* < 0.001; Fig. 4). In the second-day flowers, pollen loads were not significantly different between stigmas collected in the morning and late afternoon (*Z* = −0.415, *P* = 0.678), suggesting that pollen accumulated on stigmas mainly during the night-time. Pollen deposition during the two evenings accounted for 90.5 % of the total pollen deposition on stigmas. These results indicated that self-pollination occurred during the period of flower closure.

## Discussion

Our pollination treatments confirmed that Himalayan mayapple is SC and capable of autogamy. Our observations of flower behaviours and manipulations indicated that autogamy in *P. hexandrum* was facilitated by petal closure, rather than by pistil movement (Ma *et al.* 1997; Xu *et al.* 1997). The pistil was indeed inclined at flower opening, but it did not move towards the anthers during anthesis. Furthermore, we observed that bees were potential effective pollinators for this early flowering herb in the alpine area. Wind pollination is unlikely in the species, given that the showy flowers release sticky pollen in tetrads. Self- and cross-pollination yielded similar seed production, suggesting no pre-seedling inbreeding depression. Automatic self-pollination occurred when the petals were closed, indicating that it was delayed selfing rather than prior selfing as previously thought. Emasculated flowers yielded some seeds, suggesting that pollinator-mediated cross-pollination existed.

### Scarcity of pollinators and delayed self-pollination

Local solitary bees were occasionally seen visiting Himalayan mayapple during observations in two flowering seasons. Although terminal flowers are visible with a dark, pigmented shrunken leaf providing a contrasting background suitable for attracting pollinators (Rajkumar and Ahuja 2010), previous investigators did not observe pollinators (Ma *et al.* 1997; Xu *et al.* 1997; Rajkumar and Ahuja 2010). As an early flowering herb in the high-altitude Himalayas, nectarless *P. hexandrum* may have experienced a scarcity of pollinators. Delayed selfing is a reproductive strategy responsive to the scarcity of pollinators, i.e. plants allow earlier cross-pollination to predominate when pollinators are available (Cruden 1977; Wyatt 1983; Cruden and Lyon 1989; Lloyd and Schoen 1992). Delayed self-pollination is often accomplished through the movement of sexual organs in various species. For example, delayed self-pollination in *Hibiscus laevis* was accomplished through the progressive curling of the style branches down towards the anthers (Klips and Snow 1997). In *Aquilegia canadensis*, delayed self-pollination resulted from progressive elongation of stigmas up towards the stamens (Eckert and Schaeffer 1998). However, anthers collapsed onto stigmas in late anthesis to achieve self-pollination in *Kalmia latifolia* (Rathcke and Real 1993). In *Lupinus nanus* (Juncosa and Webster 1989) and *Mimulus guttatus* (Dole 1990), a pronounced curling of the lower stigmatic lobe facilitated self-pollination by corolla abscission. Corolla wilting has also been reported to achieve autogamy in *Pedicularis dunniana* (Sun *et al.* 2005). Flower closure in late anthesis or in unfavourable weather may bring delayed selfing in many species, but quantitative data on the process are few (Juncosa and Webster 1989; Corbet 1990; Dole 1990; Sun *et al.* 2005). Our measurements of flower movement and stigmatic pollen loads in Himalayan mayapple indicated that delayed selfing was facilitated by petals closing in the evening rather than by pistil movement. Consistent with the breeding system, low genetic diversity and remarkable population differentiation have been found in various populations of *P. hexandrum* (Xiao *et al.* 2006; Li *et al.* 2011).

### Diversification in the breeding system of the two mayapples

There is a growing body of evidence from phylogeographic study for allopatric speciation in sister species with a disjunct distribution between EA-ENA (Wen 1999; Xiang *et al*. 2000; Harrison *et al.* 2001; Qiu *et al.* 2011). A recent molecular phylogeographic study on Himalayan mayapple suggested the role of mountain glaciers in driving allopatric speciation by causing vicariant lineage divergence and acting as barriers to post-divergence gene flow (Li *et al.* 2011). It is interesting to know the process and driver of species diversification. The sister group of the two mayapples is *Dysosma* (Liu *et al.* 2002; Wang *et al.* 2007). Flowers bagged or self-pollinated in the Asian species *D. versipellis* did not produce seeds (Guan *et al.* 2010), suggesting that it was SI and incapable of automatic self-pollination. Compared with Himalayan mayapple, which is SC and in which no SI populations have been reported, American mayapple is mainly SI and for which 74 % of patches in central and northeastern Ohio were completely SI, while the rest could set fruits from selfing (Whilser and Snow 1992), and seed production depends on occasional visits by honeybees and bumblebees and is pollen limited (Swanson and Sohmer 1976; Rust and Roth 1981; Motten 1986; Laverty and Plowright 1988; Crants 2008). It is also one of the first plants to flower in spring, but it mainly occurs in the understory of the deciduous woodlands of eastern North America at 1400 m a.s.l. in the southern Appalachians, where some patches of SC individuals have been found in Oak Ridge and Tennessee (Policansk 1983), across central and northeastern Ohio (Whilser and Snow 1992) and in southeastern Michigan (Crants 2008). If populations frequently experience severe pollinator limitation, which often happens in high altitudes (e.g. Arroyo *et al.* 1982), SC individuals may have higher fitness than those that are SI. This difference may favour the spread of self-compatibility, particularly in isolated populations or in newly established habitats (Baker 1955). Why does American mayapple not evolve from SI to SC and self-pollination as in Himalayan mayapple?

The origin and spread of SC genotypes largely depend on the relative effects of inbreeding depression and fecundity differences between SC and SI genotypes (Schemske and Lande 1985). Phylogenetic analysis suggested that extant mayapples (SC) arose from SI ancestors (Liu *et al.* 2002; Wang *et al.* 2007). Self-pollination yielded a similar high seed set (86.5 %) compared with cross-pollination in Himalayan mayapple, suggesting no inbreeding depression at least at the pre-seedling stage. Genotypes with high fecundity of SC and a capability for selfing may spread in Himalayan mayapple in the elevated alpine area, resulting in a transition from SI to SC. In American mayapple, seed set from putative SC genotypes was only 10 % of the seed set from hand-outcrossing, preventing them from replacing SI genotypes (Whisler and Snow 1992). Low seed set in American mayapples was due to severe inbreeding depression, i.e. selective ovule abortion after self-pollination (Crants 2008).

A comparison of breeding systems in the two mayapples and their relatives (*Dysosma*) documented a shift from SI to SC in Himalayan mayapple (Liu *et al.* 2002; Wang *et al.* 2007). The last rapid uprising of the Himalayas began about 4–3 million years ago in the late Miocene (Shi *et al.* 1998). The divergence time estimated for the mayapple pairs (7 million years ago) pre-dated or almost co-occurred with the uplift of the Himalayas (Liu *et al.* 2002), suggesting that the shift from SI to delayed selfing occurred in the vicariant process. The pollinator fauna, in terms of the number of species and individuals, are generally depauperate with increasing elevation (e.g. Arroyo *et al.* 1982; Warren *et al.* 1988). The uplifted habit of Himalayan mayapple may result in the scarcity of pollinators in early spring. Large variations in morphology and population genetics have been recorded in Himalayan mayapple, for example several species or varieties have been proposed (Soejarto *et al.* 1979; Shaw 2009). Population differentiation of *P. hexandrum* in the Himalayan region, like the differentiation of the two mayapple species, may be attributed to geological isolation and delayed selfing. Our analysis in the two mayapples suggests that the loss of pollinators could drive the shift of breeding systems from SI to SC, consistent with the model that self-compatibility permits a species to colonize in a new habitat after long-distance dispersal (Baker 1955).

## Sources of Funding

This work was supported by the National Science Foundation of China (NSFC, no. 31030016).

## Contributions by the Authors

Y.-Z.X. conducted 2-year field work and Q.F. joined in 1 year. Y.-Z.X. and S.-Q.H. wrote the manuscript. All authors contributed in experimental design, data analysis and commented on the manuscript.

## Conflict of Interest Statement

None declared.
